# UNDERSTANDING AGE-RELATED DISPARITIES AND ENVIRONMENTAL BARRIERS IN OLDER ADULTS WITH MULTIPLE SCLEROSIS

**DOI:** 10.1093/geroni/igad104.3679

**Published:** 2023-12-21

**Authors:** Kyungmi Lee, Matthew Plow

**Affiliations:** Case Western Reserve University, Cleveland, Ohio, United States; Case Western Reserve University, Cleveland, Ohio, United States

## Abstract

Given the increasing life expectancy of individuals with multiple sclerosis (MS), the challenges faced by older adults with MS in terms of health changes and decreased physical function contribute to a potentially diminished quality of life. Despite this, our understanding of how this condition manifests in the context of aging remains limited. This study aims to examine age-related variations in fatigue, anxiety, depression, community engagement, and environmental barriers in older adults with MS, as well as to explore health disparities among older adults with MS. This secondary analysis involved 288 individuals with MS, divided into two age groups: below 60 years and 60 years and above. Aim 1 used T-tests and Aim 2 employed ANCOVA with disability adjustment. The results show that older adults with MS had higher levels of community engagement, disability, and behavioral-cognitive fatigue, reporting lower levels of anxiety, cognitive-motor fatigue, fatigue severity, and participation barriers than younger adults with MS. Noteworthy health disparities emerge, characterized by reduced community integration/participation (F=10.622, p=.001) and heightened environmental barriers (F=4.323, p=.039), even when adjusting for disability levels. The study highlights environmental barriers impeding social participation among older adults with MS, emphasizing the need to address these obstacles to enhance their quality of life. This underscores the imperative for customized interventions targeting reduced community disengagement and heightened environmental barriers faced by older adults with MS.

